# High prevalence of diarrheagenic *Escherichia coli* carrying toxin-encoding genes isolated from children and adults in southeastern Brazil

**DOI:** 10.1186/s12879-017-2872-0

**Published:** 2017-12-18

**Authors:** Liliana Cruz Spano, Keyla Fonseca da Cunha, Mariane Vedovatti Monfardini, Rita de Cássia Bergamaschi Fonseca, Isabel Christina Affonso Scaletsky

**Affiliations:** 1 Departamento de Patologia, Laboratório de Virologia e Gastrenterite Infecciosa, Centro de Ciências da Saúde, Universidade Federal do Espírito Santo, Av. Marechal Campos 1468, 29043-900, Maruípe, Vitória, Espírito Santo, Brazil; 2 Núcleo de Doenças Infecciosas, Departamento de Medicina Social, Centro de Ciências da Saúde, Universidade Federal do Espírito Santo, Vitória, Espírito Santo, Brazil; 3 Departamento de Microbiologia, Imunologia e Parasitologia, Universidade Federal de São Paulo, Vitória, Espírito Santo, Brazil; 4 Laboratório Central Municipal, Vitória, Espírito Santo, Brazil

**Keywords:** Diarrheagenic *E. coli*, Children, Adults

## Abstract

**Background:**

Diarrheagenic *Escherichia coli* (DEC) are important bacterial causes of childhood diarrhea in Brazil, but its impact in adults is unknown. This study aimed at investigating DEC among children and adults living in endemic areas.

**Methods:**

A total of 327 stools specimens were collected from children (*n* = 141) and adults (*n* = 186) with diarrhea attending health centers. Diarrheagenic *E. coli* (DEC) were identified by their virulence genes (multiplex polymerase chain reaction) and HEp-2 cell adherence patterns.

**Results:**

DEC were detected in 56 (40%) children and 74 (39%) adults; enteroaggregative *E. coli* (EAEC) (23%) was the most prevalent pathotype, followed by diffusely adherent *E. coli* (DAEC) (13%), and occurred at similar frequencies in both diarrheal groups. Atypical enteropathogenic *E. coli* (aEPEC) strains were recovered more frequently from children (6%) than from adults (1%). Twenty-six percent of the EAEC were classified as typical EAEC possessing *aggR* gene, and carried the *aap* gene. EAEC strains carrying *aggR*-*aap*-*aatA* genes were significantly more frequent among children than adults (*p* < 0.05). DAEC strains possessing Afa/Dr. genes were detected from children (10%) and adults (6%). EAEC and DAEC strains harboring genes for the EAST1 (*astA*), Pet, Pic, and Sat toxins were common in both diarrheal groups. The *astA* and the porcine AE/associated adhesin (*paa*) genes were found in most of aEPEC strains. High levels of resistance to antimicrobial drugs were found among DAEC and aEPEC isolates.

**Conclusion:**

The results show a high proportion of EAEC and DAEC carrying toxin-encoding genes among adults with diarrhea.

## Background

Diarrheal disease caused by *Escherichia coli* is a major public health concern around the world [1]. Diarrheagenic *Escherichia coli* (DEC) strains comprise the most common bacterial pathogens worldwide [2, 3]. DEC strains are classified into six groups based on clinical, epidemiological and virulence traits: enteropathogenic *Escherichia coli* (EPEC), enteroaggregative *E. coli* (EAEC), diffusely adherent *E. coli* (DAEC), enterotoxigenic *E. coli* (ETEC), enteroinvasive *E. coli* (EIEC) and enterohemorrhagic *E. coli* (EHEC) [2]. EPEC, EAEC, and DAEC show characteristics adhesion patterns (localized, aggregative and diffuse, respectively) to epithelial cells. EPEC is characterized by the presence of intimin (*eae*) gene causing attachment and effacement on intestinal epithelial cells and the bundle-forming pili (*bfp*) gene encoded in the EPEC adherence factor (EAF) plasmid. Typical EPEC is characterized by the presence of both *eae* and *bfp* genes, while atypical EPEC possess the *eae* gene alone. EAEC is characterized by the virulence factors that is present in the 60 MDa plasmid, which includes aggregative adherence factors (AAFs), the transcriptional activator *aggR,* anti-aggregation protein (*aap*) gene, and anti-aggregation protein transporter (*aatA*) gene. DAEC is characterized by the presence of Afa/Dr. adhesin genes*.* ETEC is characterized by the presence of heat labile (*elt*) and/or heat-stable (*est*) toxin genes. EIEC is characterized by the presence of an invasion plasmid, which encodes a number of genes for invasion that includes the *ipaH* gene*.* STEC is characterized by the presence of toxin genes (*stx1* and *stx2*) [3].

Diarrheal disease remains an important public health problem for children in developing areas of Brazil, including peri urban and rural areas. In these regions, the poor quality or absence of sanitization and of a clean water supply for the population introduce risk factors for the mortality and morbidity of childhood diarrhea. In a previous study conducted in the city of Vitória (same geographical region of the present investigation), DEC strains, especially EAEC, DAEC, and EPEC were found in 45% of cases of diarrhea in children from rural communities [4]. We conducted a survey of causative agents of diarrhea among children and adults living in peri urban areas of Brazil with poor hygiene and sanitization conditions.

## Methods

### Study subjects

The study was conducted between January 2008 and February 2009 in the city of Vitória, Espírito Santo. The study was part of a study with the aim of identifying risk factors for diarrhea in rural and peri urban areas with poor hygiene and sanitation conditions in southeastern Brazil [4]. Thirty-one different health centers provided stool samples. All enrolled patients (children and adults) were outpatients visiting the clinical health with acute diarrhea as reported by the physicians. The diarrhea was characterized by the occurrence of three or more loose, liquid or watery stools or at least one blood loose stool in a 24 h period [5]. The patients had no taken antibiotics in the week preceding the sampling. Clinical symptoms, including fevers, vomiting, abdominal pain, or dehydration were reported by the physicians.

Stool samples were collected and placed in Cary-Blair transport medium, and transported in iced boxes within 4 h to the laboratory at the Universidade Federal do Espírito Santo. Samples were inoculated onto the surface of MacConkey and Hektoen plates (Oxoid, Hampshire, UK) for the selection of *E. coli, Shigella,* and *Salmonella* isolates. After incubation for 24 h at 37 °C, four lactose-fermenting colonies with typical *E. coli* morphology, and two non-lactose-fermenting colonies were subjected to biochemical tests for identification. All *E. coli* strains were maintained in nutrient agar (Kasvi, Italy) slants at room temperature. Investigation of stool samples for parasites was performed by direct examination of stools after sedimentation in Lugol’s iodine solution [6].

### Detection of diarrheagenic *E. coli* by multiplex PCRs

All *E. coli* isolates were subjected to two multiplex PCRs, as previously described, with some modifications [7]. PCR1 assay contained a primer mix for the detection of the following virulence markers: *E. coli* attaching and effacing (*eae*) gene (for detection of typical and atypical EPEC), EAF plasmid (for detection of typical EPEC strains), and the antiaggregation protein transporter gene (*aatA*; previously referred to as CVD432 or the AA probe) (for detection of EAEC strains). Primers specific for the detection of DAEC Afa/Dr. (*afaB-C*) strains were subsequently included into this multiplex PCR. PCR2 assay contained primers specific for *elt* and *est.* (enterotoxins of ETEC), *ipaH* (invasion plasmid antigen H found in EIEC and *Shigella*), and *stx1* and *stx2* (Shiga toxins 1, 2 and variants of STEC). PCR1 assay identified EAEC, DAEC, and tEPEC by the presence of *eae* and *bfpA*, and aEPEC by the presence of only *eae*. PCR2 assay identified ETEC, EIEC, and STEC.

Three to six bacterial colonies from each stool sample were pooled for template DNA preparation immediately prior to PCR testing, suspended in 300 μL of sterile water, and boiled for 10 min. A 5-μL aliquot of this suspension was added to 50 μL of the PCR mixture (50 mM KCl, 10 mM Tris-HCl [pH 8.3], 1.5 mM MgCl_2_, 2 mM of each deoxynucleoside triphosphate), 1.5 U of AccuPrime *Taq* DNA polymerase, and 5 μM of each set of primers except for the *ipaH* primers, which used 10 μM. The reactions were run in a thermal cycler (model system 2400; Perkin-Elmer Corporation, Norwalk, Conn.) with the following cycling conditions: 94 °C for 5 min, 40 cycles of denaturation at 95 °C for 1 min, annealing at 58 °C (assay 1) or 50 °C (assay 2) for 1 min and primer extension at 72 °C for 2 min followed by a final extension at 72 °C for 7 min. PCR products (10 μL) were visualized after electrophoresis in 2% agarose gels in Tris-borate-EDTA buffer and ethidium bromide staining. In all assays, a mixture of DNA from the prototype EPEC E2348/69, EAEC 042, DAEC C1845, ETEC H10407, EIEC EDL1284, and STEC EDL931 strains [2] served as the positive control, while *E. coli* K-12 DH5α was the negative control [8].

All DEC strains were submitted to slide agglutination with polyvalent and monovalent antisera (PROBAC, São Paulo, Brazil) against O antigens of EPEC serogroups (O26, O55, O86, O111, O114, O119, O125, O126, O127, O128ab, O142, O158), and EHEC O157. All *E. coli* strains were kept in nutrient agar slants at room temperature.

### Detection of virulence markers by PCR

Primers and PCR conditions for detecting sequences encoding 17 putative virulence genes are described in Table 1. A DNA template was prepared by boiling a suspension of 5 colonies in 100 μl distilled water. The following *E. coli* strains where used as controls for detection of target genes: 042 (*aggR*, *aap*, *aafA, pet, astA, pic*) [9], 17–2 (*aggA*) [10], RN785–1 (*agg3A, irp2*) [11], EDL933 (*hdaA*, *chuA*), FBC114 (*sat*) [12]*, iucA* [13], C1845 (*afaE, daaE*) [14], 2787 (*aida/aah*) [15], and HSP7–1 (*paa*) [16].

**Table 1:** Primers used in polymerase chain reaction analysis

Gene	Description	Primer Sequence (5′- 3′)	PCR product	Reference
*aggR*	Transcriptional activator	CTAATTGTACAATCGATGTA ATGAAGTAATTCTTGAAT	308 bp	[37]
*aap*	Antiaggregation protein	CTTTTCTGGCATCTTGGGT GTAACAACCCCTTTGGAAGT	232 bp	[37]
*aggA*	AAF/I fimbria subunit	TTAGTCTTCTATCTAGGG AAATTAATTCCGGCATGG	450 bp	[37]
*aafA*	AAF/II fimbria subunit	ATGTATTTTTAGAGGTTGAC TATTATATTGTCACAAGCTC	518 bp	[37]
*agg-3A*	AAF/III fimbria subunit	GTATCATTGCGAGTCTGGTATTCAG GGGCTGTTATAGAGTAACTTCCAG	462 bp	[38]
*had*	AAF/IV fimbria subunit	TCCATTATGTCAGGCTGCAA GGCGTTAACGTCTGATTTCC	411 bp	[41]
*paa*	Porcine AE/associated adhesin	ATGAGGAACATAATGGCAGG TCTGGTCAGGTCGTCAATAC	357 bp	
*aida/aah*	AIDA-I adhesin	TCGATACCGAAACGCATACGCAGA ACGCCGATCGGTGATGATGAAGAT	204 bp	
*afaE*	Afa-I afimbrial adhesin	CGAAAACGGCACTGACAAG AGGCTTTCCGTGAATACAACC	230 bp	[34]
*daaE*	F1845 fimbrial adhesin	TGACTGTGACCGAAGAGATGC TTAGTTCGTCCAGTAACCCCC	380 bp	[34]
*sat*	Secreted autotransporter toxin	CTCATTGGCCTCACCGAACGG GCTGGCAGCTGTGTCCACGAG	299 bp	
*pic*	Serine protease precursor	ACTGGCGGACTCATGCTG T AACCCTGTAAGAAGACTGAGC	387 bp	
*pet*	Plasmid-encoded toxin	GACCATGACCTATACCGACAGC CCGATTTCTCAAACTCAAGACC	600 bp	
*astA*	EAST1 heat-stable toxin	CCATCAACACAGTATATCCGA GGTCGCGAGTGACGGCTTTGT	111 bp	
*chuA/ shuA*	Heme receptor	ATCTGCTGCGTCATGTTCCT GTAGTGGTCATACCTTTGAGC	1700 bp	
*iucA*	Aerobactin sintase	AGTCTGCATCTTAACCTTCA CTCGTTATGATCGTTCAGAT	1100 bp	
*irp2*	Iron chelating	AAGGATTCGCTGTTACCGGAC TCGTCGGGCAGCGTTTCTTCT	264 bp	

### HEp-2 adherence assay

*E. coli* isolates were subjected to HEp-2 adherence tests by the method originally described by Scaletsky et al. [17], with slight modifications. Briefly, monolayers of 10^5^ HEp-2 cells were grown in Dulbecco modified Eagle medium containing 10% fetal bovine in 24-well tissue culture plates (Falcon Becton Dickinson). Bacterial strains were grown statically in 2 ml of brain heart infusion for 16–18 h. The monolayers were infected with ~3 X 10^7^ bacteria (20 μl of bacterial cultures added to 1 ml of DMEM) and incubated at 37 °C for 3 h. The infected monolayers were washed with sterile PBS, fixed with methanol, stained with Giemsa stain, and examined by light microscopy for adherence pattern.

### Antimicrobial susceptibility testing

Antimicrobial susceptibility tests were performed employing the disc diffusion method on Mueller-Hinton agar, following recommendations of the Clinical and Laboratory Standards Institute [18]. One colony of each *E. coli* isolate taken from a nutrient agar culture was inoculated into 10 mL of sterile water. The resulting suspension was applied to the surface of a 14-cm plate of Muller Hinton agar (Difco) and spread evenly with a sterile cotton-tipped applicator. The plates were incubated at 37 °C for 30 min before the application of antibiotic discs. The antibiotic discs (6 mm; all obtained from Oxoid) were amikacin (30 μg), ampicillin (10 μg), amoxicillin-clavulanic acid (30 μg), cefotaxime (30 μg), choramphenicol (30 μg), ciprofloxacin (5μg), gentamicin (10 μg), imipenem (10 μg), cotrimoxazole (25 μg); tetracycline (30 μg), and trimethoprim (5 μg). The inhibition zone diameters were measured in millimeters and interpreted in accordance with manufacturers´ recommendations. *E. coli* NCTC10418 and *E. coli* K-12 C600 were used as controls.

### Statistical analysis

The statistical analyses were performed using the SPSS version 17.0 (SPSS Inc., Chicago, IL). Statistical differences were evaluated by chi-square or Fisher’s exact tests. A *p* value <0.05 was considered statistically significant.

## Results

### Subjects

From January 2008 and February 2009, a total of 327 cases of diarrhea were recruited in this study. They were divided into two groups, 141 children (< 18 years of age) and 186 adults (≥ 18 years of age), were recruited in this study. Of the 141 children, 75 (53.2%) were younger than 2 years, 49 (34.7%) were between 2 and 10 years, and 17 (12.1%) were younger than 18 years of age. Among adults, 51 (27.4%) were between 18 and 30 years, 66 (35.5%) were between 31 and 50, and 69 (37.1%) were older than 50 years of age.

### Prevalence of DEC and enteropathogens

*E. coli* (*n* = 1200) strains isolated from 280 of 327 cases were categorized into different pathotypes of DEC based on the results of two multiplex PCRs. Strains negative for DEC markers were further examined for their HEp-2 cell adherence patterns. Tables 2 and 3 show the characteristics and isolation frequency of DEC strains. DEC pathotypes were detected in 56 (39.7%) diarrheagenic children and 74 (38.8%) diarrheagenic adults. None of the DEC strains belonged to a classical EPEC serogroup. EAEC and DAEC were most common, each detected in 23% and 13%, in both diarrheal groups. Atypical EPEC (only *eae*) was more frequently detected among diarrheagenic children (5.7%) than diarrheagenic adults (1.1%). LT-ETEC was found in two diarrheagenic adults (0.7%). Mix DEC infections were detect in five patients; two of them harbored EAEC and DAEC, one harbored EAEC and aEPEC, one DAEC and EPEC, and one EAEC and ETEC. No EIEC, EHEC or STEC were detected in this study. Other enteric pathogens isolated were *Shigella* (1.2%) and *Salmonella* (0.3%). Parasites (*Ascaris*, *Giardia*, *Ancylostoma*, *Strongyloides* or *Taenia)* were detected in 7% of stool samples. Mixed infections were presented in 22 (15.6%) cases and 12 (2.9%) controls (*P* < 0.05).

**Table 2:** Distribution of diarrheagenic *E. coli* (DEC) isolated from children and adults attending health centers in Southeastern Brazil

DEC (type and genes) *n* = 130	Number (%)	% of all patients (*n* = 327)	No. of strains (%)		*p* value
			Children (*n* = 141)	Adults (*n* = 186)	
EAEC	76	23.2	32 (22.6)	44 (23.6)	0,8952
*aatA*	15 (19.7)	4.6	12 (8.5)	3 (1.6)	**0.0027**
AA phenotype	60 (78.9)	18.3	26 (18.4)	34 (18.3)	1.0000
CLA phenotype	16 (21.1)	4.9	4 (2.8)	12 (6.5)	0.1952
DAEC	42	12.8	18 (12.8)	24 (12.9)	0.3215
*afa/dr*	25 (59.5)	7.6	14 (9.9)	11 (5.9)	0.2091
DA phenotype	17 (40.5)	5.2	4 (2.8)	13 (7.0)	0.7628
EPEC	10	3.1	8 (5.7)	2 (1.1)	ND^a^
*eae*	10 (100)	3.1	8 (5.7)	2 (1.1)	ND
*eae* + *bfpA*	0	0	0	0	ND
ETEC	2	0.6	0	2 (1.1)	ND
*elt*	1 (50.0)	0	0	1 (0.5)	ND
*est*	1 (50.0)	0	0	1 (0.5)	ND
EIEC	0	0	0	0	ND
*ipaH*	0	0	0	0	
EHEC	0	0	0	0	ND
*stx1*or *stx2*	0	0	0	0	
Mixed infection	6	1.8	2 (1.4)	4 (2.2)	0.7024
EAEC + DAEC	3	0.9	1 (0.7)	2 (1.1)	ND
EAEC + ETEC	1	0.3	0	1 (0.5)	ND
EAEC + aEPEC	1	0.3	0	1 (0.5)	ND
DAEC + aEPEC	1	0.3	1 (0.7)	0	ND

^a^Not determined; *p* value in bold: significant (Fisher’s exact tests)

**Table 3:** Distribution of related-virulence genes among diarrheagenic *E. coli* (DEC) isolated from children and adults attending health centers in Southeastern Brazil

DEC group	Virulence gene	Number (%)	No. of strains (%)		*p* value
			Children (*n* = 141)	Adults (*n* = 186)	
EAEC		76			
	*aatA*	15 (19.7)	12 (8.5)	3 (1.6)	**0.0027**
	*aap*	21 (27.6)	15 (10.6)	6 (3.2)	**0.0107**
	*aggR*	20 (26.3)	14 (9.9)	6 (3.2)	**0.0181**
	*aggA*	1 (1.3)	1 (0.7)	0	ND^a^
	*aafA*	0	0	0	ND
	*agg3A*	7 (9.2)	4 (2.8)	3 (1.6)	0.4698
	*hdaA*	5 (6.6)	4 (2.8)	1 (0.5)	0.1696
	*pet*	42 (55.3)	17 (12.1)	25 (13.4)	0.7416
	*astA*	17 (22.3)	8 (5.7)	9 (4.8)	0.8040
	*pic*	31 (40.8)	14 (9.9)	17 (9.1)	0.8502
	*sat*	11 (14.5)	5 (3.5)	6 (3.2)	1.0000
	*irp2*	29 (38.1)	11 (7.8)	18 (9.7)	0.6951
	*iucA*	27 (35.5)	13 (9.2)	14 (7.5)	0.6858
	*chuA*	14 (18.4)	6 (4.3)	8 (4.3)	1.0000
DAEC		42			
	*afaB-C*	25 (59.5)	14 (9.9)	11 (5.9)	0.2091
	*afaE*	0	0	0	0
	*daaE*	0	0	0	0
	*aida/aah*	0	0	0	0
	*astA*	6 (14.2)	4 (2.8)	2 (1.1)	ND
	*pic*	3 (7.1)	1 (0.7)	2 (1.1)	ND
	*pet*	23 (54.8)	6 (4.3)	17 (9.1)	0.1251
	*sat*	11 (26.2)	3 (2.1)	8 (4.3)	0.3620
aEPEC		10			
	*astA*	10 (100.0)	8 (5.7)	2 (1.1)	ND
	*paa*	4 (40.0)	4 (2.8)	0	ND

^a^ND: Not determined; *p* value in bold: significant (Fisher’s exact tests)

### Characterization of EAEC, DAEC and aEPEC isolates

Of a total of 76 EAEC isolates, 15 (19.7%) of EAEC were *aatA* positive. EAEC *aatA*-positive strains were isolated significantly more often from diarrheagenic children than diarrheagenic adults (*p* < 0.05) (Table 2). The majority of the EAEC isolates (79%) produced the characteristic AA pattern on HEp-2 cells. Sixteen (21%) EAEC isolates produced the chain-like adherence (CLA) pattern, characterized by bacteria attaching on both coverslip and HEp-2 cell surfaces forming long chain aggregates, concomitantly with the AA pattern [19]. All EAEC isolates were tested by PCR to detect genes for the proposed EAEC virulence factors, such as Aap, AggR, AAF/I, AAF/II, AAF/III, Hda, Pet, EAST1, Pic, Irp2, IucA, and ChuA. As shown in Table 3, *pet* was the most frequently detected (55.3%) followed by *pic* (40.8%), *iucA* (35.5%), *irp2* (28.1%), *aap* (27.6%), *aggR* (26.3), and *chuA* (18.4%). One strain harbored AAF/I (*aggA),* seven strains harbored AAF/III (*agg3A*), and five strains harbored AAF/IV (*hdaA*). EAEC strains carrying the *aagR*-*aap*-*aatA* genes were isolated significantly more often from diarrheagenic children than diarrheagenic adults (*p* < 0.05) (Table 3).

There were a total of 42 DAEC, of which 25 (59.5%) harbored adhesins from the Afa/Dr. family (Table 2). DAEC strains possessing Afa/Dr. genes were detected in both children (10%) and adults (6%) groups, and none of these strains presented the adhesin-encoded genes *afaE*, *daaE* and *aida* (Table 3). All DAEC strains were tested by PCR to detect the toxin-encoding genes *astA*, *pet*, *pic*, and *sat*. As shown Tables 3, 23 (54.8%) of the strains were positive for *pet*. The *sat* gene was found in 11 (26.2%), while *astA* and *pic* were found in 6 (14.2%) and 3 (7.1%) of strains, respectively.

Atypical EPEC (only *eae*) was more frequently detected among diarrheagenic children (5.7%) than diarrheagenic adults (1.1%) (Table 2). All strains harbored the *astA* gene, and 40% of them also harbored the porcine AE-associated adhesin (*paa*) gene (Table 3). Strains were examined for adhesion to HEp-2 and none of them were adherent.

**Fig. 1:**
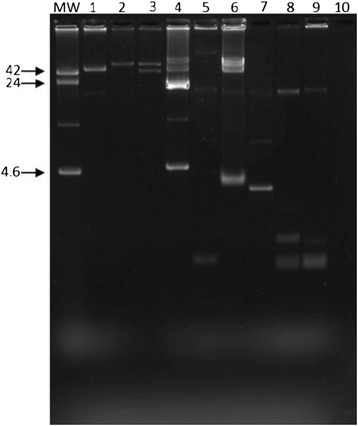
Plasmid contents of aEPEC strains isolated from children (lanes 1–8) and adults (lans 9–10). MW, R861, *E. coli* strain carrying plasmids of known molecular sizes

**Fig. 2:**
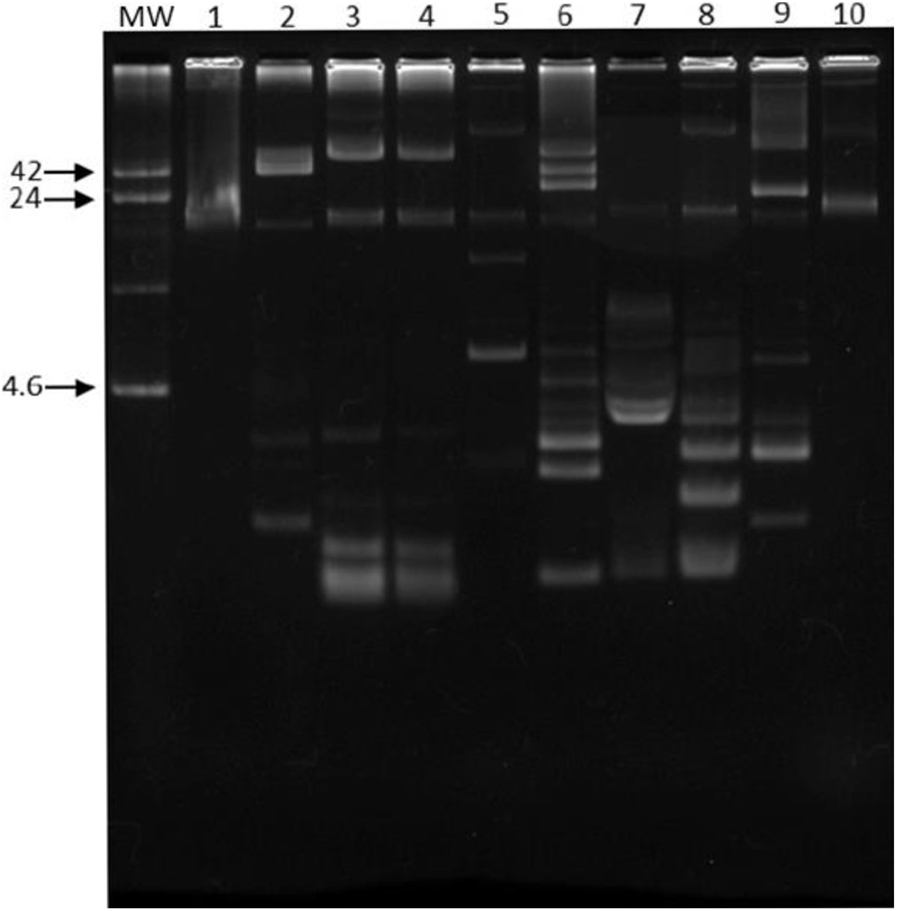
Plasmid contents of DAEC strains isolated from children (lanes 1–5) and adults (lans 6–10). MW, R861, *E. coli* strain carrying plasmids of known molecular sizes

EAEC, DAEC, and aEPEC isolates were tested for their susceptibilities to 12 antimicrobial agents (Table 4). The EAEC isolates had low frequencies of antimicrobial resistance, while high-resistance rates were found among DAEC isolates, being ampicillin, cefotaxime and cotrimoxazole the most prevalent, each detected in 75%. Half of aEPEC isolates were resistant to at least 8 antimicrobial drugs. Since it is well-known that antibiotic resistance is apparently associated with plasmids, we examined plasmid carriage of 10 strains of DAEC and aEPEC. As shown in Figs. 1 and 2, different plasmid profiles were seen after DNA extraction by alkaline lyses method [20] in DAEC and aEPEC strains isolated from both children and adults (Figs. 1 and 2).

**Table 4:** Antimicrobial susceptibility of diarrheagenic *E. coli* isolated from children and adults attending health centers in Southeastern Brazil

DEC group	Susceptibility, *n* (%)										
	AMK	AMP	AMC	CTX	CHL	CIP	GEN	IPM	SXT	TET	TIC
EAEC											
Children (*n* = 30)	1 (3.3)	0	0	0	0	1 (3.3)	1 (3.3)	0	0	1 (3.3)	0
Adults (*n* = 46)	1 (2.2)	2 (4.3)	3 (6.5)	4 (8.7)	2 (4.3)	3 (6.5)	2 (4.3)	2 (4.3)	2 (4.3)	5 (10.9)	2 (4.3)
DAEC											
Children (n = 18)	2 (11.1)	7 (38.9)	0	8 (44.4)	3 (16.7)	10 (55.5)	0	0	4 (22.2)	1 (5.5)	3 (16.7)
Adults (*n* = 24)	5 (20.8)	11 (45.8)	2 (8.3)	14 (58.3)	4 (16.7)	14 (58.3)	0	4 (16.7)	5 (20.8)	2 (8.3)	4 (16.7)
aEPEC											
Children (*n* = 8)	4 (50.0)	6 (75.0)	3 (37.5)	6 (75.0)	5 (62.5)	5 (62.5)	5 (62.5)	5 (62.5)	6 (75.0)	5 (62.5)	5 (62.5)
Adults (n = 2)	0	0	0	0	0	0	0	0	0	0	0

*AMK* Amikacin, *AMP* Ampicillin, *AMC* Amoxicillin-Clavulanic acid, *CTX* Cefotaxime, *CLO* Choramphenicol, *CIP* Ciprofloxacin, *GEN* Gentamicin, *IPM* Imipenem, *SUT* trimethoprim-sulfamethoxazole, *TET* Tetracycline, *TIC* Ticarcillin

## Discussion

Despite the abundance of reports on diarrheal disease in children under five years of age, this study is one of the few to include the identification of all six DEC pathotypes in all age individuals. Our study has shed light on the little-known issue of DEC infections in adult patients attending health centers. Adults rarely visit a health care when they have diarrhea, unless they perceive the diarrhea as being serious. We demonstrated that DEC pathotypes were commonly found in diarrheagenic adults (40%). EAEC (23%) and DAEC (13%) were the most prevalent DEC pathotypes in both diarrheal groups; whereas aEPEC strains were recovered more frequently from diarrheagenic children (6%) than from diarrheagenic adults (1%). ETEC accounted for 1.5% of DEC, and we did not find EIEC and EHEC strains, indicating their limited role in childhood diarrhea in Brazil. Our findings are in agreement with a previous study conducted in rural communities in the city of São Mateus (same geographical region of the present investigation), showing high prevalence of DEC (45%) in children with diarrhea, EAEC (21%) as the most frequent DEC, followed by DAEC (12%) and EPEC (9%) [4]. In another study, DAEC was significantly associated with diarrhea in children older than one year of age (18.3%) at the emergency room of Hospital de Pediatria in the city of Vitória [21]. Several other studies conducted in Brazil have also shown that EAEC and DAEC strains are frequently detected in children with diarrhea [22–24]. aEPEC has been increasingly reported and was recently implicated as a cause of childhood diarrhea in different urban centers of Brazil [25, 26].

The terms typical EAEC and atypical EAEC have been suggested to refer to EAEC strains harboring or lacking the regulator AggR, respectively. Some studies have demonstrated an association of typical EAEC with diarrhea [25, 27, 28]. In our study, *aggR*-positive strains were isolated significantly more often from diarrheagenic children than from diarrheagenic adults (*p* < 0.05). Interesting, AA plasmid-positive EAEC was dominant among children and AA plasmid-negative EAEC was dominant among adults. Two hypotheses would be proposed: one is that there are different routes of infection to adults and children in the study area, another is that AA plasmid-negative strains could survive adaptively in adults, though children and adults are equally infected by both AA plasmid positive and negative EAEC. Twenty percent of our EAEC *aatA*-*aggR* positive strains simultaneously harbored the *aap* gene for dispersin. There appears to be a high conservation of the *aatA-aagR*-*aap* locus in the pAA plasmid, as has been shown for the prototype 042 strains [29]. Most tEAEC did not harbor the four variants of AAFs, similarly to previous studies in Brazil [11, 22, 30].

The pathogenic mechanisms of EAEC infection are only partially understood. The varying presence of the different virulent factors indicates heterogeneity of the EAEC isolates [30]. It has been hypothesized that the combination of these genes increases strain virulence. Several different combinations of the virulence markers were found among the EAEC isolates. The most prevalent combination was *pet* and *pic*, found at similar frequencies in both diarrheal groups.

The adhesins of Afa/Dr. family have been implicated in DAEC pathogenesis. The prevalence of DAEC possessing Afa/Dr. genes in diarrheagenic children and diarrheagenic adults was similar. Germani et al. [31] demonstrated that, among DAEC strains, only those that were Afa/Dr.^+^ were found in higher frequency in diarrheic patients than asymptomatic controls. However, in some studies, DAEC Afa/Dr.^+^ strains are isolated from cases of diarrhea and controls in similar frequencies [32, 33]. The *afaE* and *daaE* (F1845) genes were not found in any DAEC strains. In our study, a significant proportion of DAEC isolates carried a gene encoding for a toxin, such as Pet and Sat. In a recent Brazilian study, DAEC *sat*-positive strains were found to be associated with childhood diarrhea [34].

The porcine AE/associated adhesin (*paa*) gene has been found in a higher frequency among aEPEC from children with diarrhea than from controls [16, 35]. In addition, the EAST1 toxin (*astA*) has been found in association with diarrheal disease among Brazilian children [36–38]. The analysis of the presence of those genes showed that all aEPEC isolates carried *astA* and 40% of them carried *paa* genes.

Our data show a high resistance rate in *E. coli* strains similar to those reported in previous studies [39, 40] and constitute a great concern in Brazil for public health. There was no significant difference in antibiotic resistance in *E. coli* strains isolated from children compared with strains from adults. Resistance to more than one antibiotic was found in approximately 60% of DAEC and aEPEC strains. The most commonly observed resistance was to ampicilin, cefotaxime and cotrimoxazole.

## Conclusion

Our results show a high proportion of DEC, where EAEC and DAEC predominate among children and adults with diarrhea. In addition, our results suggest that DEC carrying toxin-encoding genes seem to play an important role in causing sporadic diarrheal diseases in Brazil. Moreover, the findings reinforce our previous communications regarding the importance of DEC strains in childhood diarrhea in endemic areas of Brazil.
